# The relationship between substrate morphology and biological performances of nano-silver-loaded dopamine coatings on titanium surfaces

**DOI:** 10.1098/rsos.172310

**Published:** 2018-04-11

**Authors:** Weibo Zhang, Shuang Wang, Shaohua Ge, Jialong Chen, Ping Ji

**Affiliations:** 1 School of Stomatology, Shandong University, Shandong Provincial Key Laboratory of Oral Biomedicine, Jinan, Shandong 250012, People's Republic of China; 2 Stomatologic Hospital and College, Anhui Medical University, Key Laboratory of Oral Diseases Research of Anhui Province, Hefei, Anhui 230032, People's Republic of China

**Keywords:** nano-silver, dopamine, micro/nanoporous structure, smooth surface, antimicrobial activity, osteoblast-compatibility

## Abstract

Biomedical device-associated infection (BAI) and lack of osseointegration are the main causes of implant failure. Therefore, it is imperative for implants not only to depress microbial activity and biofilm colonization but also to prompt osteoblast functions and osseointegration. As part of the coating development for implants, the interest of *in vitro* studies on the interaction between implant substrate morphology and the coating's biological performances is growing. In this study, by harnessing the adhesion and reactivity of bioinspired polydopamine, nano-silver was successfully anchored onto micro/nanoporous as well as smooth titanium surfaces to analyse the effect of substrate morphology on biological performances of the coatings. Compared with the smooth surface, a small size of nano-silver and high silver content was found on the micro/nanoporous surface. More mineralization happened on the coating on the micro/nanoporous structure than on the smooth surface, which led to a more rapid decrease of silver release from the micro/nanoporous surface. Antimicrobial tests indicated that both surfaces with resulting coating inhibit microbial colonization on them and growth around them, indicating that the coating eliminates the shortcoming of the porous structure which render the implant extremely susceptible to BAI. Besides, the multiple osteoblast responses of nano-silver-loaded dopamine coatings on both surfaces, i.e. attachment, proliferation and differentiation, have deteriorated, however the mineralized surfaces of these coatings stimulated osteoblast proliferation and differentiation, especially for the micro/nanoporous surface. Therefore, nano-silver-loaded dopamine coatings on micro/nanoporous substratum may not only reduce the risk of infection but also facilitate mineralization during the early post-operative period and then promote osseointegration owing to the good osteoblast-biocompatibility of the mineralized surface. These results clearly highlight the influence of the substrate morphology on the biological performances of implant coating.

## 1. Introduction

Titanium (Ti) and its alloys are the most frequently used materials for endosseous implants in dentistry for its unique characteristics in biocompatibility, strength, fatigue and corrosion resistance [1]. Although these endosseous implants contribute significantly to the quality and effectiveness of the healthcare system with high clinical success rates, the incidence of delayed healing, implant failure and repeated surgeries is also increasing owing to biomedical device-associated infection (BAI) and lack of osseointegration at the bone-to-implant contact interface [2]. Besides operational procedures, the success rates of surgeries rely on surface characteristics of the implant such as chemical composition, surface morphology and biocompatibility. Surface morphology is an important factor determining the long-term implant stability, especially when bone quality is poor [3]. Numerous studies reported that micro-and nanoscale structure (micro/nanoporous) could mimic the hierarchically defined environment of the extracellular matrix in order to biologically instruct the behaviours of osteoblasts [4] and then facilitates biological early anchorage in the surrounding bone tissue via the ingrowth of cells into the pores owing to a higher percentage of bone-to-implant contact [3,5]. In addition, the compatible compressive modulus of micro/nanoporous Ti matches that of cancellous bone, thus reducing the stress shielding [3,6,7]. Besides, the large surface area of micro/nanoporous structures could sometimes increase over 700% compared to an ideal smooth surface, and then render the material a high loading dose of drugs and proteins [8]. But one disadvantage of porous surfaces is their enhanced ability to attach bacteria of the plaque compared with smooth surfaces, thus enhancing the risk of BAI of the peri-implant gingiva [9]. Therefore, there is particular interest in dental implantology to design surfaces that combine both porous structures to improve osseointegration and simultaneously improve the antimicrobial property to reduce the infection risk.

To reduce the infection rate, antibiotics as constitutes of implant coatings *in situ* on Ti have already been made over the past decades [10,11]. But the use of silver as an antimicrobial agent therefore receives increasing attention owing to its good stability (long-lasting release), broad antibacterial spectrum [12] and low incidence of antibiotic resistance (a major public health concern) [13]. However, in dental applications it stains dental tissue black due to the oxidation process of ionic silver [14] in the application of silver diamine fluoride and amalgam. This undesired effect has hindered its widespread use. Nano-silver (AgNPs) have recently drawn considerable attention owing to the good colour stability and large active surface areas [15–19]. To obtain a satisfactory surface containing nano-silver, many methods have been developed to apply silver on smooth surfaces, such as plasma immersion ion implantation [20], pulsed filtered cathodic vacuum arc deposition [21], and so on. Nevertheless, the major drawbacks to these methods mentioned above contain poor nano-silver/material adhesion and difficulty in controlling nano-silver size [22,23]. In addition, the need of special equipment and/or consumption of large amounts of energy and complicated multi-step procedures also limited further applications. Given these problems, a meaningful approach is to develop a simple and versatile strategy for surface modification with nano-silver. Recently, a simple method has been developed to prepare nano-silver depending on dopamine by dip-coating. Dopamine is a small-molecule mimic of the adhesive proteins of mussels and contains high concentrations of catechol and amine functional groups, which exhibit excellent affinity for most organic and inorganic surfaces [24–26]. In addition, as a reducing and stabilizing agent, dopamine has been used to reduce Ag(I) ions in the solution to form nano-silver via catechol oxidation without the need for any toxic components, leading to *in situ* formation of nano-silver on smooth surfaces coated with dopamine [22,27]. Until now, there were only a few studies about nano-silver-modified surfaces with porous structure [8,23], in which the nano-silver only deposited on the top edges, but not inside pores, so these studies did not make good use of the loading capacity of porous structures.

Another feature of interest is that dopamine induces mineralization. Zhou *et al.* reported that the dopamine coating remarkably promoted demineralized dentine remineralization, and all dentine tubules were occluded by densely packed hydroxyapatite crystals [28]. Dopamine-functionalized carbon nanotubes (CNTs) exhibit a much higher capability for hydroxyapatite crystals mineralization over pristine CNTs when incubated in a simulated body fluid (SBF) [29].

Herein, we report a simple and bioinspired approach for *in situ* growth of a monolayer of nano-silver with good dispersibility at ambient temperature not only on the top edges but also inside pores of the porous structure. Meanwhile, Ag^+^ were reduced to nano-silver on a smooth Ti surface by the same method, and then the effect of substrate morphology (porous versus smooth surface) on the physico-chemical properties and biological activity of the nano-silver-loaded dopamine coatings was investigated.

## 2. Material and methods

### 2.1. Materials

Commercial pure Ti was purchased from Baoji Non-ferrous Metal Co., Ltd. (Shanxi Province, China). Dopamine-HCl, silver nitrate, Tris base, CaCl_2_·2H_2_O, KH_2_PO_4_, NaCl, Tris-HCl and MTT were purchased from Sigma-Aldrich. Nitric acid and glutaraldehyde were purchased from Acros Organics. Fetal bovine serum (FBS), α-minimum Eagle's medium (α-MEM), Trypsin-EDTA solution, penicillin and streptomycin were purchased from Gibco. Other reagents were purchased from Sinopharm Chemical Reagent Co., Ltd.

### 2.2. Preparation and characterization of nano-silver-loaded dopamine coatings on smooth or micro/nanoporous titanium surface

Commercially available pure Ti discs were mechanically polished up to 2000 grit and sequentially by ultrasonication in acetone, ethanol and deionized water. The cleaned smooth Ti discs were denoted as *sTi.* The specimens of *sTi* were soaked in 5M NaOH solution at 60°C for 48 h, and then were soaked in deionized water at 80°C for 8 h to obtain micro/nanoporous structure and were denoted as *pTi*. The above samples of *sTi* and *pTi* were subjected to 2 mg ml^−1^ dopamine hydrochloride in Tris buffer (10 mM, pH ∼8.5) for 24 h at room temperature in the dark, then thoroughly ultra-sonicated to detach the non-attached dopamine; polydopamine coated samples were denoted respectively as *sTi/PDA* and *pTi/PDA*. Then, the dopamine-modified samples were further immersed in silver nitrate (AgNO_3_, 10 mg ml^−1^, pH ∼10) solution in an orbital shaker incubator at 80 r.p.m. and 37°C for 24 h. Next, the substrates were rinsed vigorously for 10 min in deionized water to remove the residual silver ions and were dried in vacuum for further use, the samples were denoted respectively as *sTi/PDA/AgNP* and *pTi/PDA/AgNP*.

The surface morphology and microstructure of coatings was investigated by scanning electron microscopy (SEM, Hitachi S-4800). X-ray photoelectron spectroscopy (XPS, Thermo ESCALAB 250) with an Al Ka X-ray source (1486.6 eV photons) was employed to characterize the chemical composition of substrates. Substrates were left in the introduction chamber of the XPS apparatus to outgas. Then, spectrum survey scans were performed between 0 and 1100 eV electron binding energies at pass energy of 100 eV and high-resolution spectra were performed at 30 eV for detailed scans. The system was calibrated using the C1s peak at 284.8 eV. All spectra were recorded at a take-off angle of 45 degrees. The maximum information depth of the XPS method is not more than 10 nm. Static water contact angle measurements were performed by a sessile drop method with DSA100 drop shape analysis system (DSA100, Krüss, Germany) using deionized water at ambient humidity and temperature. Five samples were measured in each group, and two separate measurements were made on each sample.

### 2.3. Mineralization of the coating

Samples were immersed in 1.5 ml of calcification solution (pH ∼7.5) and incubated in an orbital shaker incubator at 80 r.p.m. and 37°C. The composition of calcification solution contained 2.58 mM calcium (CaCl_2_·2H_2_O), 1.55 mM phosphate (KH_2_PO_4_), 180 mM NaCl and 50 mM of Tris-HCl [28]. The calcification solution was replaced every day. The samples were taken out at 7 days and rinsed vigorously for 10 min with deionized water, and then dehydrated in a graded ethanol series, followed by critical point drying and sputter-coating with gold prior to SEM analysis. The samples were denoted respectively as *sTi-M*, *pTi-M*, *sTi/PDA-M*, *pTi/PDA-M*, *sTi/PDA/AgNP-M* and *pTi/PDA/AgNP-M*.

### 2.4. Silver release

To investigate the release behaviour of Ag ions from the resulting coatings, the nano-silver modified samples were immersed in 6 ml of calcification solution each at 37°C without agitation for 30 days, and the entire volume was collected at predetermined time points, and fresh solutions were refilled accordingly. The amounts of released silver were determined through analysis of the resultant solutions by inductively coupled plasma optical emission spectrometry (ICP-OES, SPECTRO ARCOS; AMETEK, Inc., Kleve, Germany), with sample averages obtained from three separate tests.

### 2.5. Antimicrobial test

Although the microbial composition of peri-implant biofilms differs from that of periodontal disease, common bacterial species occur in both conditions [30,31]. Here, six oral microbes were tested on each surface, including *Escherichia coli* (*E. coli*), *Staphylococcus aureus* (*Sta. aureus*), *Candida albicans* (*C. albicans*), *Streptococcus mutans* (*Str. mutans*), *Actinomyces israelii* (*A. israelii*) and *Lactobacillus acidophilus* (*L. acidophilus*). The cultures of these microbial species were incubated aerobically (*E. coli*, *Sta. aureus* and *C. albicans*) or anaerobically (85% N_2_, 10% H_2_, 5% CO_2_) (*Str. mutans*, *A. israelii* and *L. acidophilus*) and were grown in brain heart infusion broth supplemented with 2% sucrose, referred to hereafter as ‘BHI’.

Fluorescence staining was used *in situ* to distinguish viable/dead bacteria cells to evaluate the antimicrobial activity for the different materials, using a LIVE/DEAD^®^ *Bac*Light™ Bacterial Viability kit (Molecular Probes, Invitrogen, Carlsbad, CA, USA). Samples were placed in a 24-well tissue culture plate and incubated with microbial suspensions at a concentration of 10^6^ CFU ml^−1^ at 37°C for different periods of time. Then, the samples were taken out and gently washed with phosphate buffered saline (PBS). The viability of the microbes on the samples was assessed using a combination dye according to the manufacturer's instruction. Each sample was stained for 15 min in darkness, and then examined under a confocal laser scanning microscope (CLSM, FluoView™ FV300, Olympus, Tokyo, Japan) at five random positions. Data analysis was performed using ImageJ. Viable microbial cells were stained green, whereas dead cells were stained red.

Zone of inhibition (ZOI) tests were carried out to determine the samples' antimicrobial effect against the strains of gram-positive and gram-negative microorganisms [32]. The samples were placed face down on a solid lysogeny broth medium agar plate surface, which was spread evenly with 20 µl of the individual test-strain solutions (10^6^ CFU ml^−1^). Zones of microbial growth inhibition (a clear zone around the sample), indicating antimicrobial activity for the obtained surfaces, were photographed after incubation for different times at 37°C

The growth curve of the microbes incubation with different samples was assayed to evaluate the samples' antimicrobial properties. The samples were incubated with 1.5 ml of different microbial suspensions at a concentration of 10^6^ CFU ml^−1^ at 37°C. One hundred microlitres of suspension was taken out for optical density (OD) measurements at 660 nm (OD_660_) using an ultraviolet--visible spectrophotometer at predetermined time points. The higher the OD value, the greater the opacity based on the turbidity of the cell suspension. A growth control with no samples was employed for each parameter.

### 2.6. Osteoblast-compatibility test

The mouse osteoblastic cell line (MC3T3-E1) was used to evaluate the attachment and proliferation of osteoblasts on the different surfaces. Cells were routinely cultured in α-MEM supplemented with 10% FBS and 1% penicillin/streptomycin in a humidified atmosphere of 5% CO_2_ at 37°C. At 80–90% confluence, cells were trypsinizated and harvested by centrifugation, and then were resuspended and seeded onto the as-sterilized specimens at a density of 4 × 10^4^ cells per sample by using 24-well tissue culture plates as the holders. The medium was refreshed every 2 days.

After incubation for 12 h, 24 h and 5 days in a cell incubator (at 37°C and 5% CO_2_), the samples were gently rinsed three times with sterile PBS to remove loosely adherent cells and then transferred to 2.5% glutaraldehyde for 4 h to fix the adherent cells. The expression of actin in cell was determined by Streptavidin Biotin FITC Complex (SABC) kit (Boster, Wuhan, China) [33]. The samples were immediately examined under a fluorescence microscope (LEICA DMRX Polar-ization microscope, Leica, Germany). Otherwise, after 12 h, 24 h and 5 day cultivation, the cell proliferation was determined by an MTT assay.

The osteoblast differentiation was evaluated by alkaline phosphatase (ALP) assay. ALP activity quantitative assay was performed using an ALP assay kit (Nanjing Jiancheng Bioengineering Institute, China) at days 4, 7 and 10 after cell seeding on different samples. Briefly, the samples were rinsed three times with sterile PBS to remove the medium. The adherent cells on each sample were lysed in 100 µl Ripa Lysis Buffer (Shanghai Sangon Biotech. Co., Ltd., China) for 30 min at 4°C. The lysates and reagent were added to a 96-well plate according to the manufacturer's instruction, followed by incubation at 37°C for 15 min and was measured at 520 nm using microplate reader. The total protein content was measured by Pierce BCA^®^ Protein Assay Kits (Thermo Scientific, Rockford, IL, USA), read at 562 nm and calculated according to a series of albumin standards. Finally, the relative ALP activity was normalized to the total protein content at the end of the experiment.

### 2.7. Statistics

All experiments were performed at least three independent times. All the quantitative data were presented as means ± standard deviations and were compared with one-way ANOVA tests to evaluate statistical significance using SPSS software. After the ANOVA analysis, a Tukey multiple comparisons test was performed to find significant differences between pairs. A *p*-value of less than 0.05 was considered statistically significant and denoted with an asterisk in the figures.

## 3. Results and discussion

**Figure 1.**
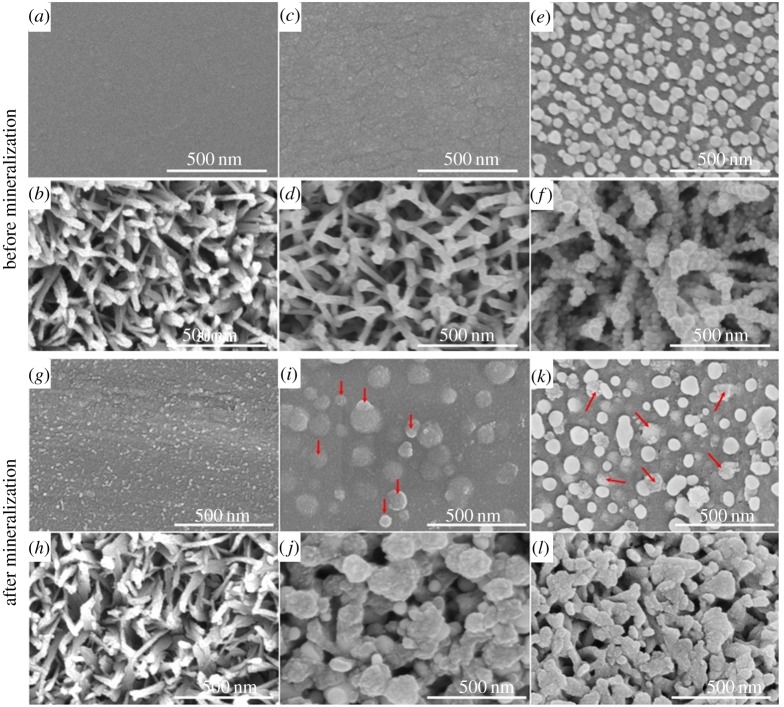
SEM images of *sTi* (*a*), *pTi* (*b*), *sTi/PDA* (*c*), *pTi/PDA* (*d*), *sTi/PDA/AgNP* (*e*), *pTi/PDA/AgNP* (*f*) and mineralized surfaces of *sTi-M* (*g*), *pTi-M* (*h*), *sTi/PDA-M* (*i*), *pTi/PDA-M* (*j*), *sTi/PDA/AgNP-M* (*k*), *pTi/PDA/AgNP-M* (*l*) after incubation for one week in the calcification solution.

Figure 1 shows the surface morphology of *sTi*, *pTi*, *sTi/PDA*, *pTi/PDA*, *sTi/PDA/AgNP*, *pTi/PDA/AgNP*, respectively. From the images, the original Ti has a smooth surface (figure 1*a*), while the surface becomes rough (figure 1*c*) after dopamine deposition, which may attribute to the polymerization of dopamine. Figure 1*e* presents the surface of *sTi/PDA/AgNP*, it can be seen that the obtained nano-silver has a spherical and irregular shape with an average diameter about 50 nm, and they are evenly distributed on the surface. As shown in figure 1*b*, the surface of the alkali-treated Ti is characterized by a uniform porous morphology with lots of struts and then polydopamine coating made the struts grew thicker (figure 1*d*). Figure 1*f* shows that the nano-silver is successfully fused in the top edges and incorporated within the pore at a much higher density. The size of the nano-silver is about 20–40 nm and their shape is spherical. Although there was more nano-silver on the *pTi/PDA/AgNP* than *sTi/PDA/AgNP*, numerous sites on the struts were not coated with nano-silver.

Luo *et al*. reported that the ratio of Ag/dopamine played a vital role in the morphological control of nano-silver [34]. With the increase of the Ag/dopamine ratio, the mean size of the nano-silver increased whereas their shapes became more irregular. Here, the micro/nanoporous structures have larger surface area coated with dopamine than the smooth surface, so the Ag (in the solution)/dopamine (on the material surface) ratio of *sTi/PDA* was greater than that of *pTi/PDA*. Consequently, the mean size of the nano-silver on *sTi/PDA/AgNP* was bigger (approx. 50 nm) than on *pTi/PDA/AgNP* (20–40 nm). Besides, catechol in dopamine could form bidentate binuclear surface complexes on Ti surfaces with covalent and ionic bond characteristics, which led to a complete firm inorganic (Ti)/polymer-matrix (polydopamine) composite surfaces. Then, the interaction between the nano-silver and polydopamine via forces of metal coordination, electron and electrostatic interactions could directly lead to the strong adherence of nano-silver to the polydopamine coatings, which is very useful and essential for practical applications [35]. Correlating these with micro/nanoporous structures, one can conclude that the surface of *pTi/PDA/AgNP* with hierarchically micro/nanoporous surface morphology was successfully developed.

**Figure 2.**
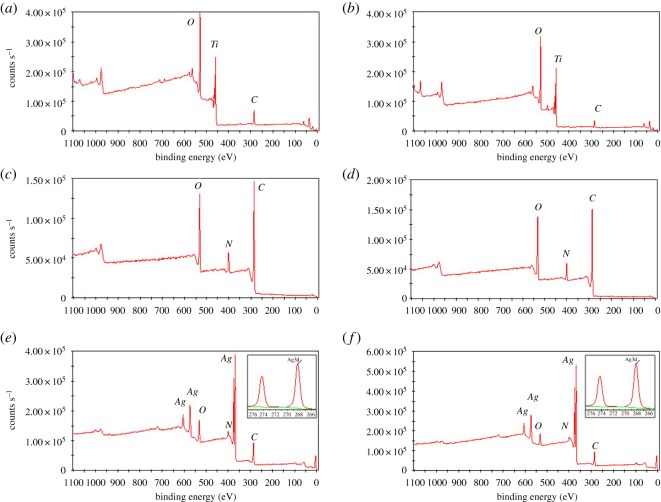
The XPS wide-scan spectra of *sTi* (*a*), *pTi* (*b*), *sTi/PDA* (*c*), *pTi/PDA* (*d*), *sTi/PDA/AgNP* (*e*) and *pTi/PDA/AgNP* (*f*). Insets present high-resolution spectra of the Ag3d peak.

The chemical composition of the surfaces at various stages of surface functionalization was determined by XPS. The XPS wide scan spectra of *sTi* (*a*), *pTi* (*b*), *sTi/PDA* (*c*), *pTi/PDA* (*d*), *sTi/PDA/AgNP* (*e*) and *pTi/PDA/AgNP* (*f*) are shown in figure 2. The presence of N1s peak (approx. 399 eV) on *sTi/PDA* and *pTi/PDA*, indicates that dopamine was successfully immobilized onto *sTi* and *pTi* (without nitrogen signal), because dopamine contains 9 atom% nitrogen. Meanwhile, the intrinsic substrate peaks of Ti2p disappeared in the *sTi/PDA* and *pTi/PDA*, also indicating that dopamine was incorporated onto the substrate materials. Silver signal was clearly present in the *sTi/PDA/AgNP* (*e*) *and pTi/PDA/AgNP* (*f*) and the inserted figures of high-resolution XPS spectrum showed two specific peaks with binding energies of 368.45 eV and 374.45 eV, which were attributed to Ag3d_5/2_ and Ag3d_3/2_ electrons of Ag^0^, respectively. The spin energy separation was identified as 6.0 eV, indicating that the silver on these surfaces was metallic Ag^0^ in nature [34]; in turn, this further supports the conclusion that nano-silver has been successfully loaded on both surfaces.

Quantitative composition of all sample types from the high-resolution spectra is shown in table 1. Compared with *sTi* and *pTi*, there was a significant decrease in the oxygen and Ti content and a significant increase in the carbon and nitrogen content on both polydopamine-coated substrates, indicating that dopamine was coated onto *sTi* and *pTi*. After nano-silver functionalization, some decrease in the oxygen, carbon and nitrogen content and a significant increase in the silver content was detected, indicating that nano-silver had incorporated onto *sTi/PDA/AgNP* and *pTi/PDA/AgNP*. Because of the large surface area of micro/nanoporous structures for nano-silver deposition, the silver content of *pTi/PDA/AgNP* (20.63%) is much higher than that of *sTi/PDA/AgNP* (14.22%). Meanwhile, the nitrogen content respectively decreased from 8.13% (*sTi/PDA*) to 6.18% (*sTi/PDA/AgNP*) and from 7.46% (*pTi/PDA*) to 6.8% (*pTi/PDA/AgNP*), thus the reduction of nitrogen content on *pTi/PDA/AgNP* (0.66%) was less than it on *sTi/PDA/AgNP* (1.95%), indicating that the exposed polydopamine on the porous surface was more than on the smooth surface.

**Table 1.** Elemental composition and ratios of different surfaces as determined by XPS.

	elements (atom%)						
samples	Ti	C	O	N	Ag	Ca	P
*sTi*	20.05	29.67	48.82	1.46	0	0	0
*sTi/PDA*	0.28	71.4	20.19	8.13	0	0	0
*sTi/PDA/AgNP*	0.11	60.49	19	6.18	14.22	0	0
*sTi/PDA/AgNP-M*	0	54.49	31.06	5.78	6.22	1.81	0.64
*pTi*	24.83	20.51	52.8	1.86	0	0	0
*pTi/PDA*	0	72.22	20.32	7.46	0	0	0
*pTi/PDA/AgNP*	0	56.81	15.76	6.8	20.63	0	0
*pTi/PDA/AgNP-M*	0	49.81	29.00	3.41	13.06	3.85	0.87

**Figure 3:**
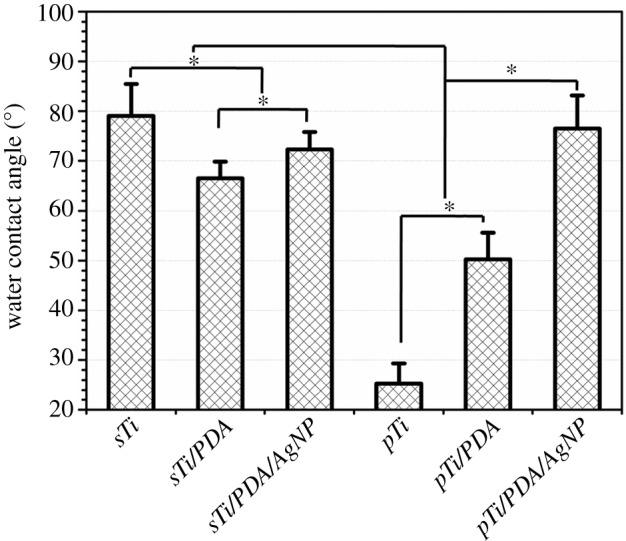
Water contact angle of different surfaces.

The hydrophilicity of the implant surface affects bio-functions such as protein adsorption and bacteria/cell adhesion. Here, static water contact angle measurements (WCA) were used to investigate the surface characteristics. As shown in figure 3, *sTi/PDA* (66.5 ± 3.4°) was more hydrophilic than *sTi* (79.0 ± 6.4°), but nano-silver made the surface hydrophobic and the WCA value of *sTi/PDA/AgNP* increased slightly to 72.3 ± 3.5°. Compared with *sTi*, alkali treatment created super-hydrophilicity of micro/nanoporous surfaces and the WCA value of *pTi* decreased significantly to 25.3 ± 4.1°. But polydopamine and nano-silver undermined the surface hydrophilicity and made the respective WCA values increase to 50.2 ± 5.4° (*pTi/PDA*) and 76.5 ± 6.7° (*pTi/PDA/AgNP*). There were no significant differences of the hydrophilicity between *sTi/PDA/AgNP* and *pTi/PDA/AgNP*. These results indirectly indicated that dopamine and nano-silver were successfully incorporated onto both smooth and porous Ti surfaces.

**Figure 4.**
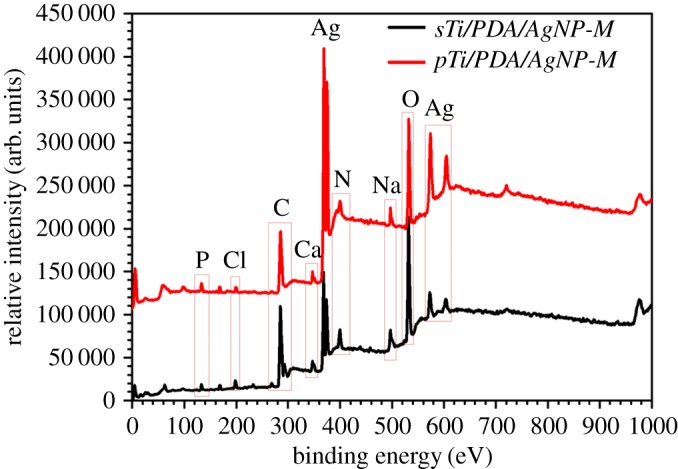
The XPS wide-scan spectra of *sTi/PDA/AgNP-M* and *pTi/PDA/AgNP-M* after incubation for one week in the calcification solution.

The mineralization potential of different surfaces was assessed by incubation with calcification solution for one week. As shown in figure 4, minerals appeared on all kinds of surfaces and had distinct morphologies. For mineralized surface of the smooth Ti (figure 1*g*), minerals were irregular grains with features of approximately 5–10 nm in diameter. Compared with *sTi-M*, minerals on the *sTi/PDA-M* (figure 1*i*) and *sTi/PDA/AgNP-M* (figure 1*k*) were spherical and far too big with features of 50–200 nm and 30–60 nm in diameter (red arrows), indicating that the resulting surface of *sTi/PDA/AgNP* could promote mineralization much more than pure Ti. Meanwhile, minerals on the micro/nanoporous Ti made the struts grow thicker (figure 1*h*). Compared with *pTi-M*, there were more minerals on the surfaces of *pTi/PDA-M* (figure 1*j*) and *pTi/PDA/AgNP-M* (figure 1*l*), which almost covered these pores, while minerals on the *pTi/PDA-M* were more than on the *pTi/PDA/AgNP-M*. These results indicate that the ability of mineralization on the nano-silver-loaded dopamine coatings is inferior to polydopamine coatings, while has an advantage over on the smooth and micro/nanoporous Ti, and the minerals on the surfaces of *pTi/PDA/AgNP-M* (figure 1*l*) were more than *sTi/PDA/AgNP-M* (figure 1*k*); one explanation for this is that the level of the exposed dopamine determines mineralization *in vitro* [28,36].

Further evidence is given by XPS. The XPS wide scan spectra of *sTi/PDA/AgNP-M* and *pTi/PDA/AgNP-M* are shown in figure 4. Both mineralized surfaces exhibited two specific peaks of Ca2p and P2p, which also indicated that mineralization happened on these surfaces. This was in good agreement with the SEM images. In an attempt to quantitatively assess the differences of mineralization between both surfaces, the high-resolution spectra of XPS was applied and the results were shown in table 1. Compared with the minerals on the *sTi/PDA/AgNP-M*, an increase in the calcium and phosphorus content was detected in the surface of *pTi/PDA/AgNP-M*. It is assumed that the micro/nanoporous surface with polydopamine and nano-silver was more effective in promoting mineralization than the smooth surface, because there was more dopamine exposed on the micro/nanoporous surface to promote mineralization *in vitro* [28,36]. Previous studies demonstrated that calcium phosphates could facilitate de novo bone formation at the bone-implant interface [37]. As such, we speculate that nano-silver-loaded dopamine coatings could show similar mineralization also *in vivo* after implantation and thus improve the bone-implant fixation.

**Figure 5.**
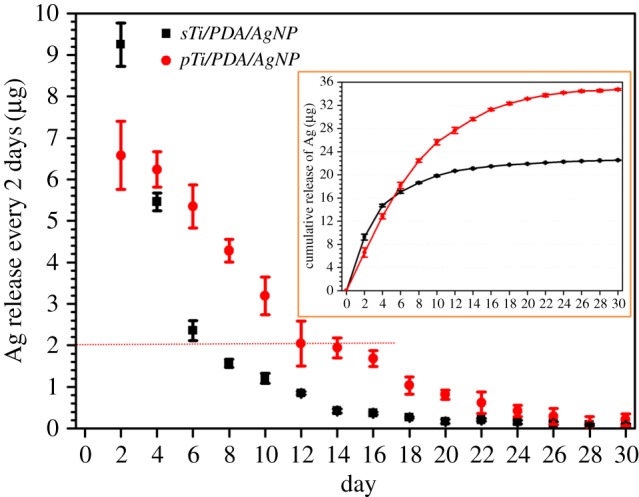
The measurement of silver release from nano-silver modified surfaces into calcification solution was sustained for 30 days. Solutions were changed every two days and silver concentration measured via ICP-OES. The inset is cumulative release of silver.

Figure 5 displays time-dependent silver release from nano-silver modified substrates in calcification solution by ICP-OES. The surface of *sTi/PDA/AgNP* provided a strongly sustained silver release and the amount of released silver every two days dropped below 2 µg within the first 14 days, meanwhile, the silver release from *pTi/PDA/AgNP* every 2 days decreased rapidly from 9.25 ± 0.42 µg to 2.35 ± 0.24 µg within the first 6 days. A rapid silver release in the early stage of incubation with saline solution (shown in inset) was also reported in other studies [22,27,38] and may be ascribed to ion exchange between the silver on the samples and cations (e.g. Ca^2+^ and Na^+^) in the saline solution. Owing to the larger surface area of the micro/nanoporous than smooth surface [8], there was more silver release from *pTi/PDA/AgNP* than from *sTi/PDA/AgNP* in the first two days. With ion exchange, parts of surfaces were coated with cation (e.g. Ca^2+^ and Na^+^), which slowed down the silver release. Besides, minerals on the *pTi/PDA/AgNP-M* reduced the contact areas between nano-silver inside pores and saline solution, and then the ion exchange at the inner surfaces of pore walls was suppressed and the silver release from micro/nanoporous surface decreased more rapidly than from smooth surface. Until 30 days of incubation, there were still 0.11 ± 0.05 µg and 0.09 ± 0.02 µg of silver release from *sTi/PDA/AgNP* and *pTi/PDA/AgNP*, showing good durability. Recent data suggests that microbial colonization on submucosal implant surfaces and the presence in sulcus fluids may occur within 10–14 days after implantation [39,40], so a sustained release of silver from nano-silver modified substrates is necessary to prevent infection during this period, but a strongly sustained silver release may induce cytotoxicity.

Biofilm formation is of interest to our study as its presence makes the treatment of implant infections extremely difficult. Although the microbial composition of peri-implant biofilms differs from that of periodontal disease, common bacterial species occur in both conditions [30,31]. Here, six oral microbes were tested on each surface, including bacterial species (Gram-negative bacterium: *E. coli* and Gram-positive bacteria: *Sta. aureus*, *Str. mutans*, *A. israelii* and *L. acidophilus*) and fungi species (*C. albicans*).

**Figure 6.**
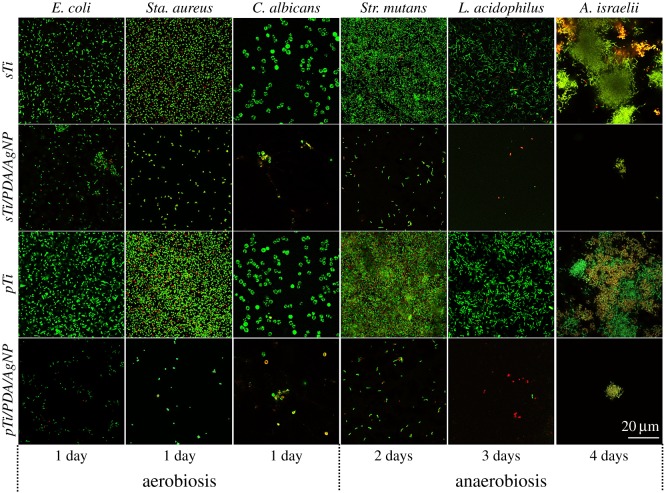
Representative confocal laser scanning micrographs of six types of oral microbes on the *sTi*, *pTi*, *sTi/PDA/AgNP* and *pTi/PDA/AgNP* after incubation at predetermined time points, visualizing live (green), dead (red) and apoptotic (yellow) cells.

The prohibition of initial bacterial adhesion onto the implant surfaces, which later could evolve into biofilms, is considered as the first and key step in combating BAI. Here, the viability of the early adherence of microbial cells was first evaluated by *in situ* live/dead fluorescent staining after cultivation on various surfaces at predetermined time points. As shown in figure 6, the surfaces of the *sTi* and *pTi* supported rapid and extensive adherence of the six types of oral microbes. The number of adhered microbes on the *pTi* was higher than on the *sTi*, indicating that the porous structure is extremely susceptible to bacterial colonization. Conversely, microbial adherence onto the nano-silver modified surfaces was suppressed by more than 95% compared with *sTi* and *pTi* over the same time period. Most of the microbial cells on the surfaces of *sTi* and *pTi* were viable (stained green) throughout the immersion period, while the dead microbial cells (stained red) and the apoptotic microbial cells (stained yellow) observed on these surfaces were mainly attributed to cell death during the microbial growth process rather than antimicrobial activity. For the nano-silver modified surfaces, the percentage of apoptotic cells (yellow) and dead cells (red) was higher than on the *sTi* and *pTi*. Only a few sparsely distributed, single viable cells were observed, indicative of the high efficiency of nano-silver conjugates in destroying the microbial cells. These results indicated that the nano-silver modified surfaces inhibited the early adherence and growth of microbes.

**Figure 7.**
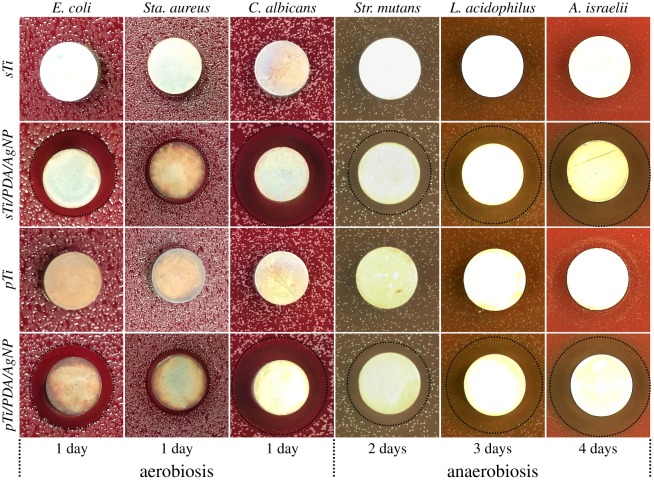
Zone of inhibition (ZOI) testing of the *sTi*, *pTi*, *sTi/PDA/AgNP* and *pTi/PDA/AgNP* against six types of oral microbes.

ZOI tests were used to investigate the ability to inhibit six types of microbial growth around samples, as shown in figure 7. After incubation at predetermined time points, only nano-silver modified samples showed clear transparent rings (black circle), indicating that nano-silver did inhibit growth or kill microbes around them.

**Figure 8.**
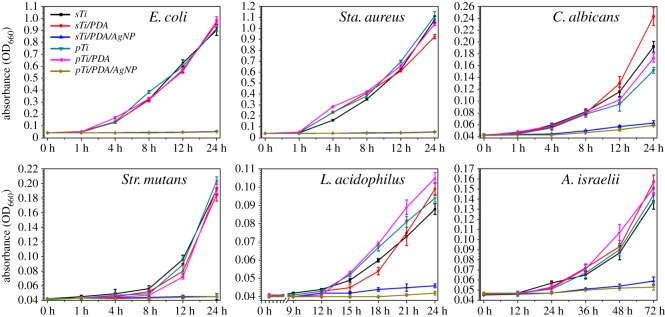
Microbial growth curves of six types of oral microbes in LB media with different samples. Comparison of the antimicrobial effect of the samples with and without nano-silver.

Previous studies revealed that nano-silver may lose their antimicrobial activity in air within 5 days [41], so their stability should also be studied. After at least one week's standing in air, the samples were incubated with microbial suspensions and microbial growth in the solution was monitored by measuring the OD at 660 nm (OD_660_). As shown in figure 8, the samples without nano-silver (i.e. *sTi*, *pTi*, *sTi/PDA* and *pTi/PDA*) did not show noticeable antimicrobial activity against the microbial growth while the nano-silver modified samples completely inhibited microbial growth, confirming that nano-silver kept their antimicrobial activity in air and prohibited microbial growth in their local environment. It is reasonable to conclude that nano-silver on both surfaces possess high and long-term antimicrobial activity owing to its high stability.

The micro/nanoporous structures render the implant extremely susceptible to bacterial colonization and subsequent biofilm formation [3]. This also was confirmed by our results, where the number of microbial cells on *pTi* was higher than on *sTi* (figure 6). Thus, it is important to prevent the microbial colonization on the porous structures. The biological rationale behind the involvement of many microbes in peri-implantitis is their capacity to efficiently attach onto implant surfaces [42], and then contribute to BAI [43]. *Staphylococcus aureus* adheres onto dental implant surfaces and contributes to 5%–40% of BAI [44]. Opportunistic fungal pathogen *C. albicans* colonizes implant sites and then raises the question over its participation in peri-implantitis [31]. *Streptococcus mutans* is implicated in modulating the virulence of bacterial biofilms in both early and late stages of peri-implantitis [45]. *Actinomyces israelii* is the dominant *Actinomyces* species isolated from implanted devices [46]. Besides, microbes from dental plaque around the implant can migrate toward the apex of the implant and subsequently develop peri-implantitis [47]. *Escherichia coli* can be detected in subgingival plaque of periodontitis patients [48] and *L. acidophilus* plays a major part in progression of plaque accumulation [49]. The most successful strategies to diminish the risk of peri-implantitis is to maintain the implant surface in contact with oral tissues as free of microbes as possible and then to release the antimicrobial compounds to inhibit microbial growth around the implant [50]. Therefore, the incorporation of nano-silver onto Ti surfaces with subsequent silver release is a sound strategy to prohibit BAI. The studies reported that the majority of biofilm formation occurs during the early post-operative period [39,40], so a rapid silver release to keep high silver content around the implant during the first few days (figure 5) is necessary. Here, the nano-silver was incorporated onto substrates to prevent microbial adherence on them (figure 6) and then a rapid silver release could inhibit microbial growth around the implant and prohibit microbial migration from dental plaque near the implant (figures 7 and 8), suggesting that peri-implantitis by BAI may be prevented. Previous research indicated that nano-silver may lose its antimicrobial activity, when exposed to air for 5 days [41,51] due to oxidation and/or aggregation, but our nano-silver could maintain its antimicrobial activity at exposure to air for one week (figure 8), probably because dopamine as a reducing agent stabilizes the nano-silver [34,52]. Antimicrobial tests demonstrate no significant differences between *sTi/PDA/AgNP* and *pTi/PDA/AgNP*, indicating that the disadvantage of a porous structure, which was susceptible to bacterial colonization and subsequent biofilm formation, may well be overcome by nano-silver coating.

**Figure 9.**
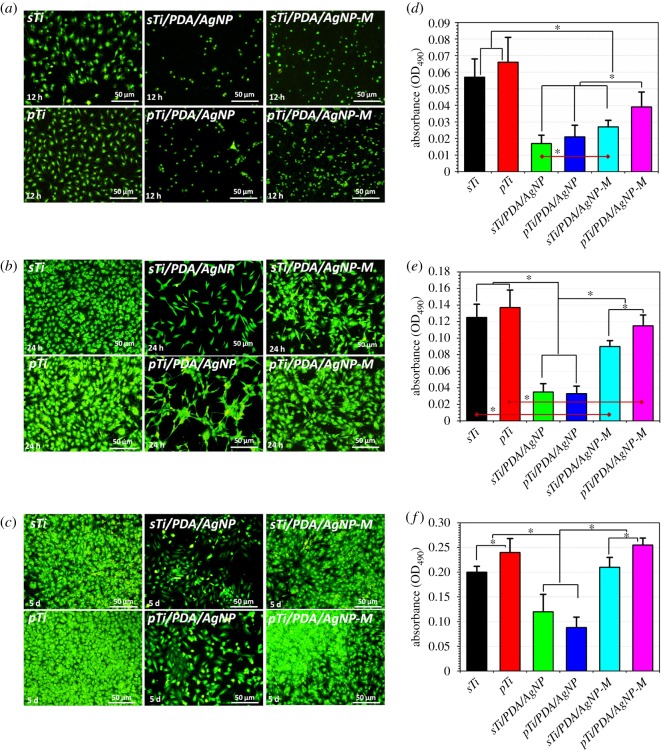
Immunofluorescent images of cytoskeletal actin for osteoblast (MC3T3-E1) on the different surfaces after 12 h (*a*), 24 h (*b*) and 5 day (*c*) culture. The quantitative analysis of osteoblast cultured on the different surfaces for 12 h (*d*), 24 h (*e*) and 5 days (*f*), measured by MTT assay.

The effects of nano-silver-loaded dopamine coatings on mouse osteoblastic cell line (MC3T3-E1) attachment and proliferation were investigated through cytoskeletal actin staining and MTT tests after 12 h, 24 h and 5 days of culture, as displayed in figure 9. In the first 12 h, adherent cells on the *sTi* and *pTi* had higher numbers and larger adhesion area than other surfaces, and exhibited favourable spreading (figure 9*a*). Meanwhile, nano-silver postponed the adhesion process and almost all adherent cells on *sTi/PDA/AgNP* and *pTi/PDA/AgNP* were near-round and did not spread, indicating that both surfaces were not favourable for the osteoblast attachment. Compared with *sTi/PDA/AgNP* and *pTi/PDA/AgNP,* there were more cells adherent on both mineralized surfaces of *sTi/PDA/AgNP-M* and *pTi/PDA/AgNP-M*. In particular, adherent cells on *sTi/PDA/AgNP-M* were inferior to *pTi/PDA/AgNP-M* in terms of quantity and morphology, indicating that the mineralized surfaces of *pTi/PDA/AgNP-M* had good cytocompatibility. Upon 24 h in culture, round cells were rarely detectable on all surfaces (figure 9*b*). Compared with cells on *sTi* and *pTi* with sub-confluent, cells on *sTi/PDA/AgNP* and *pTi/PDA/AgNP* were still detached and spread out with an unhealthy spindle osteoblastic shape, indicating that the surfaces of *sTi/PDA/AgNP* and *pTi/PDA/AgNP* were not favourable for the osteoblast growth. Meanwhile, adherent cells on *sTi/PDA/AgNP-M* and *pTi/PDA/AgNP-M* were superior to *sTi/PDA/AgNP* and *pTi/PDA/AgNP* in terms of quantity and morphology with a healthy spindle shape, indicating that minerals indeed improved cytocompatibility. The adherent cells on *pTi/PDA/AgNP-M* was more than on *sTi/PDA/AgNP-M*, indicating that the mineralized surface of *pTi/PDA/AgNP-M* was more suitable for osteoblast growth. After 5 days of culture (figure 9*c*), cells still did not cover the surfaces of *sTi/PDA/AgNP* and *pTi/PDA/AgNP*, while cells on the other surfaces were completely confluent, especially for *pTi* and *pTi/PDA/AgNP-M*, indicating that the nano-silver modified surfaces were not favourable for osteoblast proliferation but this nature could be improved after mineralization, especially for *pTi/PDA/AgNP-M*.

The quantitative analysis of cells adhered on the different surfaces was performed by MTT assay and shown in figure 9*d–f*. The OD values on the *sTi* and *pTi* were significantly higher than on the *sTi/PDA/AgNP* and *pTi/PDA/AgNP*, indicating that the nano-silver-loaded dopamine coatings exhibited cytotoxicity. With silver release and surface mineralization, the osteoblast-compatibility of both surfaces was significantly improved. The MTT metabolism on *pTi/PDA/AgNP* was higher than on *sTi/PDA/AgNP*, indicating that the mineralized surface of *pTi/PDA/AgNP-M* had better osteoblast-compatibility. After 5 days, the MTT metabolism on *pTi/PDA/AgNP-M* was highest among all of these surfaces, also indicating that this surface had excellent osteoblast-compatibility.

**Figure 10.**
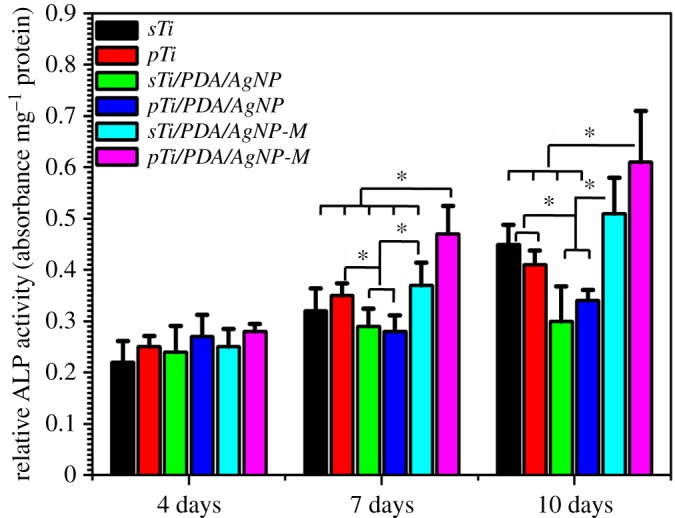
ALP quantitative assay of MC3T3-E1 on different samples at days 4, 7 and 10.

Besides the initial cellular attachment and proliferation, the subsequent ALP activity at 4, 7 and 10 days was measured as an early hallmark for osteogenic differentiation potential of MC3T3-E1. The results of quantitative assay showed that the normalized ALP activity for all samples increased over time throughout the assay period (figure 10). There were no significant differences between different samples at 4 days, but osteoblast on *sTi/PDA/AgNP-M* and *pTi/PDA/AgNP-M* presented higher ALP activity than other samples at 7 days and 10 days, indicating that the mineralized surfaces could induce osteoblast differentiation, one explanation for this is that calcium and phosphorus accelerated osteogenesis [53]. In addition, osteoblast on *pTi/PDA/AgNP-M* presented highest ALP activity, also indicating that this mineralized surface could induce more osteogenic differentiation.

After metal implants are introduced into the human body, bacterial infections should be prevented in the first place. The nano-silver on the surfaces and a strongly sustained silver release could prevent microbial infections during the early post-operative period. Though some studies reported that the nano-silver-modified surfaces had no significant cytotoxicity [32,38,54], here, both nano-silver-loaded dopamine coatings were not favourable for the osteoblast attachment, proliferation and differentiation. After incubation with the calcification solution for one week, silver release and surface mineralization significantly enhanced osteoblast-compatibility of both nano-silver-loaded dopamine surfaces. So we speculate that both surfaces could adsorb mineral elements from body fluid to achieve mineralization during the early post-operative period and then the mineralized surfaces could be favourable for the osteoblast attachment, proliferation and differentiation. Besides, the mineralized surface with porous structure (*pTi/PDA/AgNP-M*) had better cytocompatibility than the smooth surface (*sTi/PDA/AgNP-M*). We assume that less release of silver and higher calcium and phosphorus content and porous structure (figure 4) are the main causes.

## 4. Conclusion

The nano-silver-loaded dopamine coatings were successfully prepared on micro/nanoporous or smooth Ti surfaces by immersion in a dopamine solution, followed by immersion in a silver nitrate solution. Compared with the smooth surface, small size of nano-silver and a high silver content was obtained on the micro/nanoporous surface, owing to the large surface area of the porous structure. More mineralization occurred on the coating with the micro/nanoporous structure than on the smooth surface, which led to a more rapid decrease of silver release from the porous Ti surface. Both nano-silver-loaded dopamine coatings did inhibit microbial colonization on them and growth around them. There was no significant difference in antimicrobial properties between both coatings. The coatings on both surfaces were not favourable for the osteoblast attachment, proliferation and differentiation, but these disadvantages were overcome by surface mineralization, especially in micro/nanoporous surfaces (*pTi/PDA/AgNP-M*). Therefore, the nano-silver-loaded dopamine coating on micro/nanoporous structures may not only reduce the risk of infection but also facilitate mineralization for further osteointegration of Ti implants. These results clearly revealed the significance of the effect of substrate morphology on physico-chemical characterization and biological functions of the coating. This simple, facile, and environmentally friendly technique is therefore believed to have great potential for clinical application.

## Data accessibility

This study's dataset is available at Dryad: http://dx.doi.org/10.5061/dryad.365f3 [55].
